# The PAICE project: Integrating health and health equity into UK climate change policy

**DOI:** 10.12688/wellcomeopenres.23431.1

**Published:** 2025-01-15

**Authors:** Michael Davies, Charlie Dearman, Rosemary Green, Andrew Haines, Clare Heaviside, Filiz Karakas, Sudheer Kumar Kuppili, Susan Michie, James Milner, Gemma Moore, David Osrin, Silvia Pastorino, Giorgos Petrou, Irene Pluchinotta, Charles Simpson, Phil Symonds, Catalina Turcu, Sarah Whitmee, Ke Zhou, Nici Zimmermann

**Affiliations:** 1 UCL Institute for Environmental Design and Engineering, University College London, London, UK; 2 Department of Public Health, Environments and Society, London School of Hygiene & Tropical Medicine, London, England, UK; 3 Department of Population Health, London School of Hygiene & Tropical Medicine, London, England, UK; 4 Clinical, Educational and Health Psychology, University College London, London, England, UK; 5 Institute for Global Health, University College London, London, UK; 6 Bartlett School of Planning, University College London, London, England, UK

**Keywords:** climate change action; health and health equity; policy; systems; integration, transdisciplinary research.

## Abstract

This paper announces a new initiative - the research project *Policy and Implementation for Climate & Health Equity* (PAICE), which aims to investigate the complex systemic connections between climate change action, health and health equity, for translation of evidence into policy and practice in the UK. Using transdisciplinary approaches, PAICE will: (1) co-develop a programme theory and linked monitoring and evaluation plan, (2) work with the UK Climate Change Committee (CCC) and the Greater London Authority (GLA) using system dynamics to analyse national and local policy opportunities, (3) build an integrated model of the effects of these policies on population health, health equity and greenhouse gas emissions, (4) apply the findings to the CCC monitoring framework and GLA policy development, and (5) use the programme theory to help evaluate achievement of PAICE processes and objectives. If successful, PAICE will have helped to establish a systems capability to (i) monitor whether Government plans are on track to deliver their climate targets and associated health impacts and (ii) understand how relevant policy and implementation approaches could be enhanced.

## Plain Language summary

This paper describes the plans for a new initiative – the research project *Policy and Implementation for Climate & Health Equity* (PAICE). PAICE aims to investigate the complex interactions between climate change action, health and health equity for translation of evidence into policy and practice in the UK. The World Health Organization defines health equity as “the absence of unfair and avoidable or remediable differences in health among population groups” and addressing this vital issue is a key aspect of PAICE. PAICE is a collaboration between teams of academics at UCL and LSHTM who will work closely with the UK Climate Change Committee (CCC) and the Greater London Authority (GLA) to analyse national and local policy opportunities. If successful, PAICE will have helped to establish a capability to (i) monitor whether Government plans are on track to deliver their climate targets and associated health impacts and (ii) understand how relevant policy and implementation approaches could be enhanced.

## Background

The required changes crucial to addressing closely coupled climate and health challenges ( Haines *et al.*, 2009) go well beyond those achieved so far by any country. In terms of climate targets, the UK has been a world leader. It was the first country to set a legally binding target to reduce greenhouse gas emissions ( HMG, 2008) and the first to set a Net Zero target ( *The Climate Change Act 2008 (2050 Target Amendment) Order 2019*). However, despite important achievements, it is failing in much of its implementation ( CCC, 2024). Acceleration of healthy, sustainable development requires transformative ( Crane *et al.*, 2021), scaled-up solutions developed through (i) ‘co-creation’ of research and implementation with local stakeholders, (ii) balancing local and global responsibilities, (iii) emphasising health and health equity as primary objectives, and (iv) harnessing the worldwide experience of transdisciplinary research and practice. It requires a profound understanding of the system structure behind the lack of progress in implementation. A systemic lens is needed to identify the connections between challenges in different sectors and the consequent actions that can, by transforming systems, contribute to meeting multiple objectives efficiently and effectively. Evidence-based approaches to inform policy ^1^ development and ultimately implementation are vital but challenging, as is an understanding of the distributional impacts of interventions for health equity.

Assembled in response to these challenges, PAICE will focus on national and local policy and implementation in the UK, and engage with other countries to exchange knowledge. The team comprises two UK universities (UCL and LSHTM), the UK Climate Change Committee (CCC) and the Greater London Authority (GLA). The CCC is an influential independent statutory body established under the Climate Change Act 2008. Its role is to advise the UK Government on emissions targets and report to Parliament on progress made in reducing greenhouse gas emissions and preparing for climate change. The GLA is the devolved regional governance body of Greater London. It has two political branches: an executive Mayor and the 25-member London Assembly, which serves as a means of checks and balances on the Mayor.

## Aims, objectives and anticipated impacts

PAICE aims to investigate the complex systemic connections between climate change action, health and health equity, and what these mean for translation of evidence into policy and practice in the UK. Taking a transdisciplinary approach, we have co-developed the following research objectives through discussions with the research team and policy partners:

1. **Understand and evaluate** the value of the PAICE programme in delivering transdisciplinary systems research and guiding policy formulation and implementation.

2.  Enhance the CCC’s annual/biannual sectoral mitigation and adaptation **monitoring frameworks ^2^**, explicitly incorporating health and health equity.

3. **Integrate** the CCC’s mitigation and adaptation monitoring frameworks using a systems approach.

4.  Feed into and enhance the CCC **recommendation-making processes** for both the annual/biannual progress reports and the 5-yearly carbon budget/Climate Change Risk Assessment (CCRA) advice reports.

5. **Translate** the research by working with the GLA to adapt the CCC mitigation and adaptation monitoring frameworks for regional and local application.

PAICE has short-, medium- and long-term anticipated impacts:

*- Short-term:* PAICE will have informed the annual and bi-annual CCC progress reports to the UK Parliament that make detailed recommendations for every government department. PAICE will have worked with the GLA to help develop monitoring frameworks, informed by the CCC’s national frameworks but tailored for local application, for the sectors of housing, energy, food and transport.

*- Medium-term:* PAICE will have informed detailed CCC advice reports on climate change mitigation and adaptation, which directly inform UK Government policy.

*- Long-term:* PAICE will have helped to establish a systems capability to (i) monitor whether Government plans are on track to deliver their climate targets while recognising associated health and health equity impacts and (ii) understand how policy and implementation approaches could be enhanced (including via the incorporation of systems thinking tools and analysis). The project will also have served as a potential model for similar initiatives worldwide, informing debates on mitigation and adaptation policy.

## Research questions

PAICE is a complex programme with multiple layers (wider systems change, programme level aspirations and individual work package aims). We will be switching between a focus on work packages and the programme, whilst thinking about the wider context in which the programme sits. We considered these levels when thinking about our research questions. As a result, PAICE has established three sets of research questions that address the system, programme and work package levels. For example:

*- At system level:* How does the CCC integrate evidence and findings from PAICE into its progress reports and recommendations?

What criteria are used to assess the relevance, reliability and applicability of this evidence?

*- At programme level:* What are the shared priorities among stakeholders regarding climate change mitigation/adaptation, health equity and reductions in greenhouse gas emissions?

How can these priorities inform policy development and, ultimately, implementation?

*- At work package level:* What are the cross-sectoral impacts of specific policy measures ^3^ on population health, health inequalities and greenhouse gas emissions?

## Underpinning principles

Central to the PAICE project are the underpinning principles of systems thinking, transdisciplinary working and academic-policy engagement.

### Systems thinking

The PAICE project builds on systems thinking and system dynamics modelling approaches. This is in response to the fact that climate change interventions and their impact on health involve complex systems characterised by multiple and often conflictual priorities, non-linearities, interdependences, feedback relationships and inherent delays, making it difficult for decision-makers and policy measures to anticipate the consequences of their actions (adapted from Richardson (2011)). By incorporating both qualitative and quantitative systems modelling, PAICE will provide a deeper understanding of rich system structure, enabling an iterative learning process of all the parties to replace a reductionist view of climate change with a holistic long-term dynamic view, reinventing our policies and institutions accordingly (adapted from ( Sterman, 2006)).

More generally, systems thinking is the ability to see the world as a complex system and to represent and assess its dynamic complexity ( Sterman, 2000). System dynamics is a computer-aided formal modelling approach enabling a comprehensive understanding of complex dynamics systems ( Forrester, 1969), by analysing the system behaviour resulting from the interactions of its components over time ( Sterman, 2000). It achieves this by incorporating feedback loops among the different components as well as supporting the assessment of the potential impacts of system disturbances ( Meadows *et al.*, 1972).

Building on the team’s previous research ( Coletta *et al.*, 2024a; Coletta *et al.*, 2024b; Dianati *et al.*, 2019; Eker *et al.*, 2018; Macmillan *et al.*, 2016; Pluchinotta *et al.*, 2021; Pluchinotta *et al.*, 2022; Pluchinotta *et al.*, 2024a; Pluchinotta *et al.*, 2024b; Prioreschi *et al.*, 2024; Shrubsole *et al.*, 2019; Zimmermann *et al.*, 2021), the project takes a systems approach to addressing this complexity, clarifying the issues that need to be addressed, investigating their causes, co-developing solutions, co-producing knowledge and supporting policy measures implementation. Adopting a systems perspective in PAICE can help minimize unintended consequences by fostering a comprehensive understanding of the issues and their potential causes ( Stave *et al.*, 2019), including different systems’ views ( Pluchinotta *et al.*, 2022) and integrating different sources of knowledge ( Pluchinotta *et al.*, 2024a).

PAICE acknowledges that meeting climate targets is impossible without changes in mental models and behaviours of people and institutions. While setting up the systems approach to support this is challenging, PAICE will leverage the science of behaviour and behaviour change and integrate its theoretical frameworks in the systems activities, to support the analysis of barriers and enablers for successful implementation of decision, making recommendations on levers for actions ( Michie *et al.*, 2014). For behaviour to change, there needs to be not only capability (knowledge and skills), but also motivation and opportunity, physical and social. This is represented by the Capability, Opportunity and Motivation (COM-B) model which acts as a guiding framework; by understanding behaviour in its context, one can identify interventions and policies most likely to be effective ( Michie *et al.*, 2011).

### Transdisciplinary research (TDR)

PAICE also draws on transdisciplinary research ideas in response to real-world problems and characterized by interactions between stakeholders in participatory problem solving ( Hansson & Polk, 2018; Lawrence *et al.*, 2022; Rigolot, 2020, p. 20). Stokols *et al.* (2013) define transdisciplinarity as “ *scholars and practitioners from both academic disciplines and non-academic fields working jointly to develop and use novel conceptual and methodological approaches that synthesize and extend discipline-specific perspectives, theories, methods, and translational strategies to yield innovative solutions to particular scientific and societal problems.*”

Transdisciplinary research integrates both researchers from unrelated disciplines and non-academic participants (here, the CCC and GLA) in pursuit of a common goal, with the intention that the collaboration produces new knowledge and theory. It involves both interdisciplinarity and co-creation (co-design of research and co-production of knowledge) with actors outside academia ( OECD, 2020). We previously built on the work of Stokols *et al.* (2013), to help develop a new model ( Pineo *et al.*, 2021) for transdisciplinary health research that entails iterative stages of co-learning, pre-development, reflection and refinement, conceptualisation, investigation and implementation. The practical translation of transdisciplinary working within the project is to integrate diverse perspectives, to include participatory, behavioural science and social research methods, and to elicit knowledge from stakeholders (see Dianati *et al.* (2019); Moore *et al.* (2023); Pineo *et al.* (2021)).

### Academic-policy engagement

There is a growing body of literature investigating how researchers engage with and influence policy processes, ultimately seeking to ensure evidence use in policymaking ( Cairney & Oliver, 2020; Hopkins *et al.*, 2021). However, there is often a disconnect between the ‘producers’ of scientific evidence and the ‘users’, which can lead to barriers to evidence use ( Cairney & Oliver, 2017). A variety of engagement strategies are emerging to broker relationships between academic and policy practices; for example, research–practice partnerships, policy placements, fellowships, areas of research interest (ARIs), and policy-engaged research projects. Best and Holmes (2010) describe three generations of ‘exchange’ in how knowledge and policy action can inform and interact with each other: (1) linear, (2) relational, and (3) systems approaches. They argue that there has been a shift from simple processes of disseminating knowledge (often through one-way modes of communication such as policy briefs), to strategies that acknowledge the roles of systems and relationships at the interface between research and policy processes. Relational and systems approaches are perceived to influence the potential for research impact and overcome barriers to evidence use within the policy process, such as availability and timely access to relevant research, as well as the clarity and relevance of research ( Oliver *et al.*, 2014). Transdisciplinary research projects conceived as influencing and making policy require sufficient time, sustained engagement and expertise in knowledge brokerage.

## The need for a programme theory

The components of the project’s research and the evaluation of its impact will be shaped by a programme theory developed through a participatory process of discussion among the PAICE team to ensure the input of a broad range of perspectives and shared understanding. The programme theory aims to explain how the project’s collaborative research will work to achieve its desired effects, following structures and processes through a chain of linkages ( Rogers, 2008; Stein & Valters, 2012). A key requirement is that the group spend sufficient time surfacing assumptions ( Rogers, 2008; Stein & Valters, 2012; Weiss, 1995), understanding epistemologic and linguistic differences between participants, and articulating the links between activities and outcomes ( Stame, 2004).

Although the phases of transdisciplinary research overlap and cycle, the programme theory will encompass four general phases derived from existing conceptualisations ( Lawrence *et al.*, 2022; Pineo *et al.*, 2021): formation (identifying the issue, enlisting the team), formulation (building collaboration, defining and structuring the problem, aims, objectives and methods), investigation (collecting and analysing data that integrates disciplines and background and new knowledge) and translation (producing publications, tools and resources, integrating the findings and communicating and disseminating them).

The programme theory also provides a framework for evaluation by guiding the evidence needed to assess (1) whether and how PAICE achieves its aims, and (2) whether it improves transdisciplinary and cross-sectoral understanding and work. The evaluation framework will address questions about the success of response to known challenges of transdisciplinary research. These can be categorised as challenges to framing the problem (different ways of thinking ( Pohl, 2011), limited options for solutions and evolving expectations), challenges to working together (insufficient mutual understanding ( Pohl, 2011), insufficient integration and variation in methodological standards), challenges to organisational dynamics (insufficient timescales ( Scholz & Steiner, 2015a), unbalanced ownership of the problem across the partners and discontinuous participation), and societal challenges (insufficient legitimacy of the group and research, real-world constraints and insufficiently conclusive findings).

The programme theory will also address the challenges inherent in transdisciplinary research by structuring the project to manage the complexity of the partnerships, emphasising open communication about goals, ways of working and expectations, clarifying the added value for participants ( Scholz & Steiner, 2015b), and continuously reviewing disciplinary diversity, interaction and integration.

## Research design and methods

**Figure 1.**
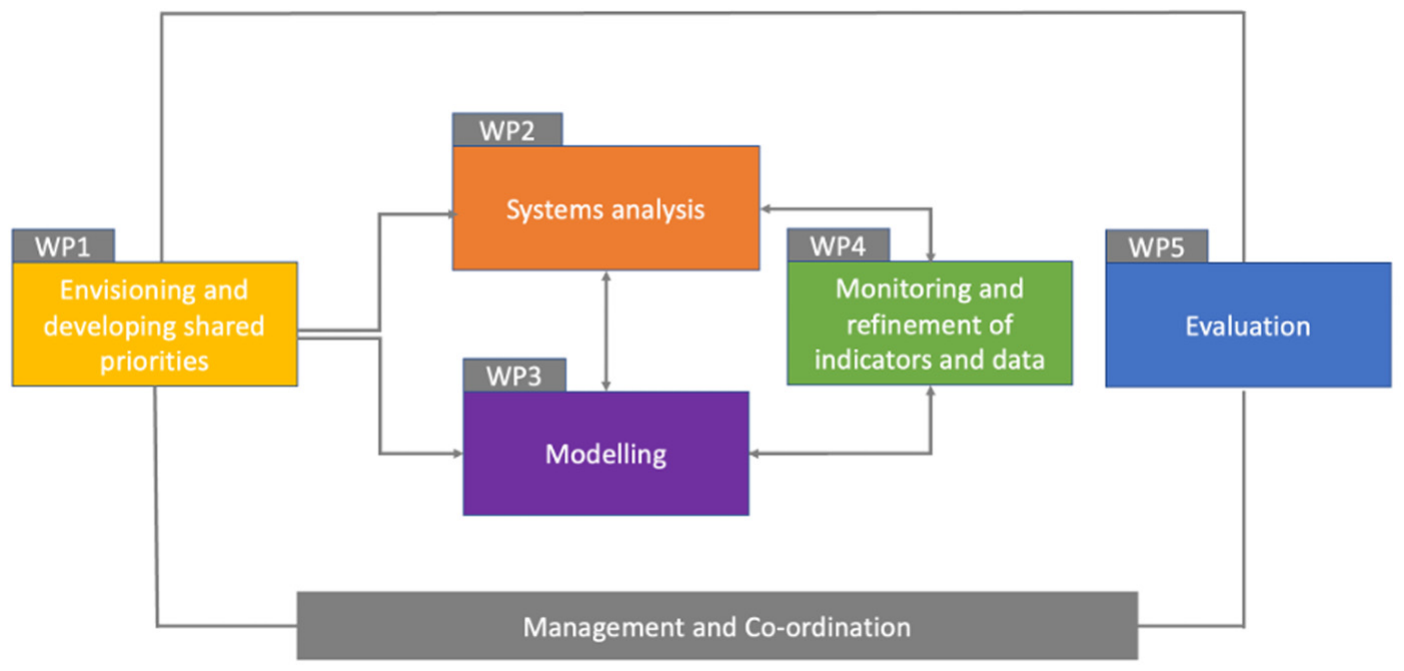
PAICE Work Packages (WPs).

PAICE is organised into five work packages (WPs) – see Figure 1. The expected outputs and outcomes from each WP are shown in Table 1.

**Table 1.** PAICE WP outputs and outcomes.

Work package	Expected Outputs	Expected Outcomes
**Envisioning and** **developing shared** **priorities (WP1)**	- PAICE Programme Theory - MELP	- Shared understanding of the system and vision among contributors - Clear articulation of the changes the programme plans to achieve (and expected pathways for change) - Visual representation of the programme, a tool to communicate with other stakeholders - Agreed transdisciplinary conceptual framework
**Systems analysis** **(WP2)**	- Policy measures and stakeholder mapping - Systems models for assessing cross-sector impacts of policy measures and of factors influencing system change	- Decision processes informed by provision of model outputs on multiple impacts - Understanding of barriers to and facilitators of transformative change and of trade-offs/spillovers - Identification of key implementation pathways and actors - A set of policies rated along different dimensions of “implementability” and equity
**Modelling (WP3)**	- Synthetic population model to estimate impacts of policies on health and health inequalities - Integrated estimates of the impacts on GHG emissions and health of cross-sectoral policy options	- Enhanced understanding of the ancillary effects for health and equity (the co-benefits and unintended consequences) of climate mitigation and adaptation policy options - Decision processes informed by provision of model data on multiple impacts
**Monitoring and** **refinement of** **indicators and** **data (WP4)**	- Tailored data to inform and improve the CCC monitoring framework - London-specific data to inform GLA policy priorities - Development of appropriate indicators of progress for health impacts	- An integrated understanding of progress in mitigating and adapting to climate change and the impacts on health - A gap analysis examining data availability and quality for monitoring and assessing progress against policy objectives, a map of current data and indicators used by the CCC and GLA and a recommended set of data and indicators which could be developed to better track progress
**Evaluation (WP5)**	- Relevant materials, recommendations and reports - Website and dissemination materials	- Generation of knowledge for theory and practice in policy- engagement and influence - Sharing of learnings with international partners

## Envisioning and developing shared priorities (WP1)

As noted above, the PAICE partners will co-create a programme theory describing how the programme is understood to “work” in terms of its anticipated changes and the processes of change (what the PAICE team think will happen and how). It will guide the programme to develop, reflect and learn about the conditions needed for effective climate change mitigation, adaptation and health policies. The theory will be co-developed iteratively through workshops with the PAICE team, interviews with researchers and partners, and small group discussions. It will reflect our transdisciplinary approach, linking the programme to wider social and political contexts, and the roles of stakeholders in achieving change. Our past experience has shown that the process of co-producing a programme theory facilitates alignment of perspectives and reaching a shared understanding and vision amongst contributors ( Moore *et al.*, 2021). The PAICE programme theory will provide framing for the project’s evaluation (WP5), leading to a Monitoring, Evaluation and Learning Plan (MELP).

## Systems analysis (WP2)

WP2 will identify current and planned climate policy measures in the housing, energy, food and transport sectors, starting from the CCC monitoring maps. This will inform the modelling of the relevant GHG emissions, health and health equity impacts, working closely with WP3.

PAICE will develop a systems perspective regarding policy measures using participatory system dynamics - a framework and process for gathering input from a wide range of actors and experts ( Pluchinotta *et al.*, 2022) and to coproduce knowledge on the system under consideration ( Pluchinotta *et al.*, 2024a).

The process involves several iterative steps, beginning with stakeholder mapping and conducting interviews with individual stakeholders to gather their systems’ perspectives and priorities on climate policy measures ( Salvia *et al.*, 2022). This is followed by building qualitative and quantitative system models (causal loop diagrams and stock-and-flow models) to understand the main interconnections between the different sectors and health, and the factors that accelerate or hinder system change; thereby assessing the power of measures to transform systems. Participatory workshops will review the cross-sector interactions and policy measures. These workshops will also refine the understanding of different stakeholders’ priorities and perspectives on factors affecting systems change and support policy measure implementation. Systems models will be used to collaboratively explore the cross-sector effects of suggested policy measures, including unintended consequences, trade-offs, synergies, and leverage points ( Coletta *et al.*, 2024a; Dianati *et al.*, 2019; Pluchinotta *et al.*, 2021). The process also involves analyzing barriers to and enablers of the successful implementation of decisions and policy measures, informed by behavioural science ( Michie *et al.*, 2014) and its theoretical frameworks ( Michie *et al.*, 2011), and will make recommendations on levers for action.

## Modelling (WP3)

Informed by WP2 and building on methods and expertise developed by the team’s previous work ( Alae-Carew *et al.*, 2022; Brousse *et al.*, 2022; Eustachio Colombo *et al.*, 2021; Milner *et al.*, 2023; Symonds *et al.*, 2021), PAICE will build a UK-wide population model to test the effects of policy options, allowing estimation of distributional impacts (e.g. health inequalities), targeting of policies for different population subgroups and overlay of multiple policy actions spanning different sectors. Drawing on good practice guidance ( Hess *et al.*, 2020), our modelling will allow integrated characterisation of each set of sectoral policies (housing, energy, food and transport) in terms of health and health inequalities, as well as contribution to GHG target reductions and adaptation needs, taking account of practical constraints and likely uptake. Through engagement with the CCC and the GLA, the model will allow us to test policy and implementation options and inform the development of new indicators ^4^ to track progress (in WP4).

A preliminary version of the model has already been successfully set up through CUSSH (an international research programme on the complex systemic connections between urban development and health – see Davies *et al.* (2021)), and has been used to perform initial assessments of climate actions identified for different sectors ( Milner *et al.*, 2020; Symonds *et al.*, 2021). It entails generating individual members of a large synthetic, geographically-stratified population that is representative of the true UK population in terms of age, sex, socio-economic structure, environmental and health behaviours, GHG emissions and health (mortality and morbidity risks also related to age, sex and socio-economic characteristics). The characteristics of the population will be derived from evidence published by the UK Office of National Statistics, population surveys and routine statistics coupled with sectoral data on housing, transport patterns and dietary data from routine surveys, including the English Housing Survey, the National Travel Survey and the National Diet and Nutrition Survey.

## Monitoring and refinement of indicators and data (WP4)

PAICE will assess progress in both policy development and implementation through identification and refinement of data and indicators relevant to the monitoring and reporting of climate, health and equity outcomes in the UK from net zero policies. The CCC monitoring framework is intended to identify where changes are needed to stay on track for the UK’s climate action targets. PAICE is uniquely placed to contribute to its continued development and ongoing use via the integration of the impact of cross-sectoral policies on GHG emissions, health and health equity. PAICE will work with the CCC to enhance their new monitoring framework ( CCC, 2022) and populate the sectoral models of change, ensuring that they include health and health equity indicators and data to assess health outcomes in an integrated way.

PAICE will also work with the GLA to assess the implications of downscaling the data and indicators from the national level to allow monitoring and reporting of outcomes at the regional level within the Greater London region.

Building on the team’s previous work in the Pathfinder Initiative ( Whitmee *et al.*, 2024) and the WinWindow project, PAICE will identify relevant datasets on health and equity and develop appropriate indicators with which to monitor the impact on health of climate mitigation and adaptation policies.

The outputs from the system dynamics modelling in WP2 and the emissions and population health modelling in WP3 will feed into the work of WP4. In a cyclical manner, the work of WP4 will also inform the workshops and engagement in WP2 to allow us to iteratively recommend needed adjustments and additions to policies to deliver objectives to agreed trajectories of change.

## Evaluation (WP5)

To provide informed and ongoing decision-making as part of the design, development and delivery of PAICE, evaluation and reflection processes will be embedded within and throughout the programme ( Pineo *et al.*, 2021). These processes will draw upon a developmental evaluation approach that supports innovation and adoption, providing rapid, real-time, focused feedback. We will focus on understanding the programme’s evolution, how different disciplines and practitioners work together best, and how to address cross-sectoral challenges. An evaluation working group will coordinate activities, review information and feed learning into future programming.

The programme theory generated in WP1 will provide the framework to guide the evaluation and identify specific milestones that can build on and be informed by the findings of the programme. Activities such as participatory workshops, interviews and observations will provide opportunities for reflection and collaborative learning alongside generating knowledge for theory and practice in policy-engagement and influence.

PAICE will also contribute to knowledge on the implementation of climate action policies, synthesising and drawing out transferable learning and recommendations for international partners.

## Summary

Our ambition in the PAICE project is to develop evidence on the connections between climate change policy measures, health and health equity to help accelerate transformative actions, focusing primarily on the UK. To generate this evidence we are developing new, integrated modelling methods and ways of engaging with those influencing and making policy via a framework which recognises the complex systems nature of the problem. The aspiration is to use such improved knowledge to accelerate action at scale and pace. Our programme theory sets out what actions we will take and where we expect to contribute to change. We will use the programme theory as the basis of a detailed evaluation of the PAICE approach. We will, through systems and relational approaches to engagement, share the findings to maximise the likelihood that our work will inform an urgently needed new model of action-oriented research in this area.

## Ethics approval

Ethics approval (ID:20231213_IEDE_STA_ETH) was given on 13/12/23 by the University College London BSEER (Bartlett School of Environment, Energy and Resources) Research Ethics Committee.

## Disclaimer

The views expressed in this article are those of the authors. Publication in Wellcome Open Research does not imply endorsement by Wellcome.

## Data Availability

No data are associated with this article.
