# Supplementary material for: The PAICE project: Integrating health and health equity into UK climate change policy
Source: Wellcome Open Res. 2025 May 30;10:14. Originally published 2025 Jan 15. [Version 2] doi: 10.12688/wellcomeopenres.23431.2 (PMC11947659; doi:10.12688/wellcomeopenres.23431.2)
Supplement: Peer review history

# Reviewer report: Reviewer response for version 2

**DOI:** 10.21956/wellcomeopenres.26522.r124565

**Published:** 2025-06-10

**Authors:** Annabelle Workman

**Affiliations:** 1 Melbourne Climate Futures, University of Melbourne, Melbourne, Australia

I am satisfied that the authors have sufficiently responded to our feedback and can confirm I am comfortable with the status of the article being updated

Does the article adequately reference differing views and opinions?

Yes

Are all factual statements correct, and are statements and arguments made adequately supported by citations?

Yes

Is the Open Letter written in accessible language?

Yes

Where applicable, are recommendations and next steps explained clearly for others to follow?

Partly

Is the rationale for the Open Letter provided in sufficient detail?

Yes

Reviewer Expertise:

climate change action; policy; health and health equity; transdisciplinary research

I confirm that I have read this submission and believe that I have an appropriate level of expertise to confirm that it is of an acceptable scientific standard.

# Reviewer report: Reviewer response for version 2

**DOI:** 10.21956/wellcomeopenres.26522.r124567

**Published:** 2025-06-10

**Authors:** Muriel Mac-Seing

**Affiliations:** 1 University of Montreal, Montreal, Québec, Canada

I read the revised version and the answers, and I’m happy with them.

Does the article adequately reference differing views and opinions?

Partly

Are all factual statements correct, and are statements and arguments made adequately supported by citations?

Yes

Is the Open Letter written in accessible language?

Partly

Where applicable, are recommendations and next steps explained clearly for others to follow?

Partly

Is the rationale for the Open Letter provided in sufficient detail?

Yes

Reviewer Expertise:

health equity, policy analysis, inclusive climate action, disability,  intersectionality, sexual and reproductive health and rights, mixed methods, qualitative research, participatory approaches

I confirm that I have read this submission and believe that I have an appropriate level of expertise to confirm that it is of an acceptable scientific standard.

# Reviewer report: Reviewer response for version 1

**DOI:** 10.21956/wellcomeopenres.25841.r119033

**Published:** 2025-03-26

**Authors:** Muriel Mac-Seing

**Affiliations:** 1 University of Montreal, Montreal, Québec, Canada

The *PAICE project: Integrative health and health equity into UK climate change policy* is relevant and necessary, given the global climate crisis we are all facing. PAICE aims to “investigate the complex systemic connections between climate change action, health and health equity, and what these mean for translation of evidence into policy and practice in the UK.”.

**Here are some comments:**

- **Health equity:** Given the overarching goal of addressing health equity at the systems level, it is essential to systematically incorporate health equity considerations in all key levels of the proposed systems thinking framework. For example, what feedback loop would enable PAICE to address promptly (in)equities that would have emerged among different population groups in situations of vulnerability and marginalisation? Is climate-related health equity systematically addressed when analysing methods and activities designed to achieve research objectives through the work packages? Providing more information on this would help clarify what the main key health inequity issues (in the background section) in the context of climate change are and how health equity is embedded throughout the project and how it is operationalized.
- **Co-development:** The project is ambitious and requires the social and political buy-in of various stakeholders (researchers, decision-makers, policy-makers, sectoral actors, etc.). Co-development of the research objectives is indeed appropriate. Could you provide more information on the co-development processes, who was involved (sectors, levels, disciplines), and whether representatives of citizens and civil society organisations participated?
- **Participatory approach:** Given the health equity objective of the project, how will citizens and civil society organisations, alongside researchers and policy-makers, have a voice in the five work packages?
- **Different population sub-groups:** In the work package 3, it is mentioned that “PAICE will build a UK-wide population model to test the effects of policy options, allowing estimation of distributional impacts (e.g. health inequalities), targeting of policies for different population subgroups…”. Can you provide more information on how different populations, such as women, children, old people, people with disabilities, homeless people, migrants, and other minority groups are affected by the current climate crisis in the UK and how they will be involved in the project? How accessible will the project’s work package be to them in terms of information, communication, and physical accessibility?
- **Systems:** Housing, energy, and transport are key determinants of health. They are also the sectors selected for PAICE. Could you provide more insight into why the health system (including healthcare and public health) was not included? While the health system aims to improve the health of individuals, it can also contribute to population health inequities, particularly for population groups in situations of vulnerability and marginalisation. Furthermore, it would have been important to analyse how the health system directly contributes to more comprehensive climate adaptation measures.

Does the article adequately reference differing views and opinions?

Partly

Are all factual statements correct, and are statements and arguments made adequately supported by citations?

Yes

Is the Open Letter written in accessible language?

Partly

Where applicable, are recommendations and next steps explained clearly for others to follow?

Partly

Is the rationale for the Open Letter provided in sufficient detail?

Yes

Reviewer Expertise:

health equity, policy analysis, inclusive climate action, disability,  intersectionality, sexual and reproductive health and rights, mixed methods, qualitative research, participatory approaches

I confirm that I have read this submission and believe that I have an appropriate level of expertise to confirm that it is of an acceptable scientific standard, however I have significant reservations, as outlined above.

# Response

**Published:** 2025-04-23

**Authors:** Michael Davies

**Affiliations:** UCL IEDE, University College London Bartlett Faculty of the Built Environment, London, England, UK

**Revision Notes** ***The PAICE project: Integrating health and health equity into UK climate change policy*** We would like to thank the reviewers for their very positive, constructive and helpful comments, which we have carefully considered and addressed in the revision of the paper. We provide a new version of the paper with all changes made in response to the suggestions of the reviewers tracked. Please note that Reviewer 2 (R2) - Melanie Boeckmann, University of Bremen, Bremen, Germany) did not suggest any changes and so the changes noted in the table below relate only to the suggestions of Reviewer(s) 1 (R1 - Annabelle Workman Arthur Wyns, University of Melbourne, Melbourne, Australia) and Reviewer 3 (R3) - Muriel Mac-Seing, University of Montreal, Montreal, Québec, Canada). Below, we outline our response to each of the comments. **Reviewer comments** **Response to the comments** ***1. General comment (R1):*** *Since none of the authors are affiliated with CCC or GLA, it would be good for the article to (i) clarify who in the CCC and GLA would be your main collaborators, (ii) confirm in writing that the CCC and GLA have agreed to collaborate on the project, and (iii) the role the CCC and GLA will play in each respective Work Package.* (i) In the CCC we are working closely with members of the Secretariat and in the GLA with the Public Health and Climate teams. We have now noted this in the text (ii) We have now specifically noted in the text that the CCC are collaborating on the project. (iii) We have noted that we will be engaging with the CCC and GLA with regards to each work package to plan, undertake and implement work in each WP. ***2. General comment (R1):*** *The 5 research objectives could be more clearly linked to the 5 Work Packages. Currently the subchapters describing each work package describe the activities that will be undertaken, but do not clearly link back to the 5 main outputs/research objectives (at least not always or not in sufficient detail). * We have now indicated the links to the relevant work packages. ***3. General comment (R1):*** *Given the project’s focus on developing appropriate indicators with which to monitor the impact on health of climate mitigation and adaptation policies, it would be good to add a sub-chapter or a few paragraphs on the theoretical underpinnings of the health co-benefits of mitigation measures.* *It also seems to me to be a very significant step forward to go from - on the one hand - the published findings of the Pathfinder Initiative indicating a relative lack of indicators for health co-benefits in certain sectors, to - on the other hand - an extensive set of indicators across various sectors that can be incorporated into monitoring frameworks. It would be good to expand on how PAICE will manage to deliver this progression within the constraints of the project.* We have now noted the increasing attention to this issue and provide a reference to a relevant example of our previous work where the issue is discussed in detail.         We have added some relevant text in this section to address this suggestion. ***4. R1:*** *“In terms of climate targets, the UK has been a world leader”: I would suggest clarifying this sentence by adding a verb, i.e.  ‘In terms of setting climate targets’. As you rightfully point out further down, the UK should perhaps not be considered a champion when it comes to implementing climate targets.* The word ‘setting’ has been inserted. ***5. R1:*** *“Acceleration of healthy, sustainable development”. Suggest adding ‘climate-resilient’ to this list, which is crucial especially in relation to climate and health.* ‘Climate resilient’ added ***6. R1:*** *“It requires a profound understanding of the system structure behind the lack of progress in implementation”: I would recommend leaving this sentence out, or as a minimum to provide further clarification. The current wording implies the lack of progress is caused solely by the inherent system in which society operates.* We have replaced the word ‘behind’ with ‘contributing to’. ***7. R1:*** *“ Evidence-based approaches to inform policy development and ultimately implementation”: Suggest adding ‘monitoring of progress’ as a third function here, especially given the objectives of the PAICE program.* ‘…monitoring of progress’ now added. ***8. R1:“*** *anticipated impacts: Short-term”: given your objective to translate the research by working with the GLA (and the need for this research to take place first before it can be translated) would it be more appropriate to label the work with the GLA as medium-term or even long-term?* This objective has now been moved to the medium term. ***9. R1:*** *“ Underpinning principles: Academic-policy engagement”: this is a helpful theoretical overview. This paragraph would benefit from a few sentences on the way PAICE hopes to strengthen this academic-policy engagement (similarly to the first paragraph in your ‘Systems thinking’ subchapter). For example, how will you be working with the CCC? What will this engagement look like when you translate research with the GLA?* A few relevant sentences have been added in this section. ***10. R1*** *: “ Underpinning principles: Transdisciplinary research”: When you describe ‘actors outside academia’, it might be worthwhile to point out that in the case of this project, that will include policymakers.* We have now noted the inclusion of policymakers. ***11. R1:*** *“WP2 will identify current and planned climate policy measures in the housing, energy, food and transport sectors, starting from the CCC monitoring maps”: could you please provide a reference or more information on what the CCC monitoring maps contain? As someone who is not very familiar with the CCC’s work, I currently struggle to fully understand the starting point of WP2 (ie what data is already available, versus what will have to be modelled).* In this section links are now provided to the CCC monitoring framework reports. ***12. R1*** *:"PAICE will also work with the GLA": this paragraph merits further detail as to how exactly the collaboration with the GLA will take place.* In this section we have now noted a planned secondment from the academic team to the GLA. ***13. Background (R1):*** *Is there an opportunity to briefly expand on the challenges of evidence-based policy development and the impact on health (in)equity? For those not working in the climate and health space, it might not be clear that poorly designed policies can exacerbate and entrench existing health inequities, which is an important point as it underpins the rationale for using indicators and monitoring frameworks to evaluate and track progress.* We have drawn upon the wording of this very useful suggestion to add some relevant text to this section. ***14. Background (R1):*** *Is there an opportunity to briefly explain the siloed approach not only within adaptation and mitigation interventions (i.e. between sectors), but also between these two climate responses? This will help to substantiate the need for an integrate mitigation and adaptation monitoring framework.* Some text has been added to this section in response to this suggestion. ***15. Systems analysis (WP2) (R1):*** *Who will the participatory workshops be held with to review cross-sector interactions and policy measures?* Some text has been added to this section to clarify that these workshops will involve the CCC. ***16.Evaluation (WP5) (R1):*** *“Activities such as participatory workshops, interviews and observations will provide opportunities for reflection and collaborative learning…” Is there any mechanism through the GLA (or CCC) that involves representatives from marginalised or at-risk populations? I.e. is there an avenue for those who experience health inequities to input into the project in any way? Assuming community engagement is out of scope for the project, it might be worth flagging this – or clarifying if it is a distinct process undertaken by GLA/CCC via other means.* Some text has been added to this section to address this suggestion. Including the voices of citizens and representatives of minoritised groups is important, given our emphasis on inequalities, and is something we want to strengthen in current and future work. We have secured multiplier funding to pilot a programme of community involvement in a local authority in London. A series of workshops will bring together residents, non-government organisations, researchers and creatives to discuss questions around the relevance and meaning of climate change mitigation and adaptation. ***17. Health equity (R3):*** *Given the overarching goal of addressing health equity at the systems level, it is essential to systematically incorporate health equity considerations in all key levels of the proposed systems thinking framework. For example, what feedback loop would enable PAICE to address promptly (in)equities that would have emerged among different population groups in situations of vulnerability and marginalisation? Is climate-related health equity systematically addressed when analysing methods and activities designed to achieve research objectives through the work packages? Providing more information on this would help clarify what the main key health inequity issues (in the background section) in the context of climate change are and how health equity is embedded throughout the project and how it is operationalized.* We have inserted the following text in the ‘Systems analysis (WP2)’ section: *We will use our systems approach to help map existing climate change related health inequities and how climate action might impact on them.  For instance, we know that indoor exposure to  pollutants and heat is unequally distributed with respect to, for example, ethnicity, age and income and climate action has the potential to improve (or worsen) such inequities (Cole et al., 2024). WP2 will work closely alongside WP3 (population modelling including the health impacts of air quality and heat) to further our understanding of such  issues. We expect some aspects that relate to vulnerability and equity to be represented in feedback structure, and additionally, it is an issue that will inform the direction of much of our modelling.*   Cole, R., Simpson, C. H., Ferguson, L., Symonds, P., Taylor, J., Heaviside, C., Murage, P., Macintyre, H. L., Hajat, S., Mavrogianni, A. and Davies, M. (2024). ‘A population-level framework to estimate unequal exposure to indoor heat and air pollution’. *Buildings & Cities*, 5 (1). doi: 10.5334/bc.425. ***18, 19 and 20 (R3)*** ***Co-development):*** *The project is ambitious and requires the social and political buy-in of various stakeholders (researchers, decision-makers, policy-makers, sectoral actors, etc.). Co-development of the research objectives is indeed appropriate. Could you provide more information on the co-development processes, who was involved (sectors, levels, disciplines), and whether representatives of citizens and civil society organisations participated?* ***Participatory approach:*** *Given the health equity objective of the project, how will citizens and civil society organisations, alongside researchers and policy-makers, have a voice in the five work packages?* ***Different population sub-groups:*** *In the work package 3, it is mentioned that “PAICE will build a UK-wide population model to test the effects of policy options, allowing estimation of distributional impacts (e.g. health inequalities), targeting of policies for different population subgroups…”. Can you provide more information on how different populations, such as women, children, old people, people with disabilities, homeless people, migrants, and other minority groups are affected by the current climate crisis in the UK and how they will be involved in the project? How accessible will the project’s work package be to them in terms of information, communication, and physical accessibility?* We have grouped our responses to these three comments together.   The co-development work to date has involved the CCC, GLA and the PAICE team and this is now being widened to involve other groups. Please see the response above to **point 16** as to how PAICE is involving representatives from marginalised or at-risk populations.     A recent report for the CCC addressed in detail the contextual issues of health equity with respect to climate change (Munro et al., 2020). That report noted: *“Communities that are already disadvantaged are among the most vulnerable to the effects of systemic shocks and extreme events and climate change has the potential to widen existing health inequalities within the UK. Moreover, some hazards are unavoidable due to climate change that is already ‘locked-in’ by existing concentrations of greenhouse gases in the atmosphere, and therefore adaptation and resilience must be considered in tandem with the mitigation of climate change.”*   We have now included this quote from the  report in the background section and refer the reader to that publication for further detail. Also please note the mention our population health model in the response to **point 17**.   Munro, A., Boyce, T. and Marmot, M. (2020). *Sustainable health equity: achieving a net-zero UK*. Institute of Health Equity. Available at: https://www.theccc.org.uk/publication/ucl-sustainable-health-equity-achieving-a-net-zero-uk/ (Accessed: 14 April 2025). ***Systems:*** *Housing, energy, and transport are key determinants of health. They are also the sectors selected for PAICE. Could you provide more insight into why the health system (including healthcare and public health) was not included? While the health system aims to improve the health of individuals, it can also contribute to population health inequities, particularly for population groups in situations of vulnerability and marginalisation. Furthermore, it would have been important to analyse how the health system directly contributes to more comprehensive climate adaptation measures.* We agree with the reviewer as to the importance of the health system. A detailed modelling treatment of the health system is outside the scope of the current PAICE project due to resource limitations. However, we note that it does figure importantly within the CCC monitoring framework and thus will form part of our overall systems analysis work. 

# Reviewer report: Reviewer response for version 1

**DOI:** 10.21956/wellcomeopenres.25841.r119040

**Published:** 2025-02-26

**Authors:** Melanie Boeckmann

**Affiliations:** 1 University of Bremen, Bremen, Germany

Dear colleagues

 it is of course interesting to hear about a large scale new project and its potential. I believe the project is well described, the planned work is made transparent, and the open letter can support others seeking info to know to whom reach out

 Differing views are not presented as per the report questions for review, but I don't think presenting criticisms of a planned project is the task of the project authors? I would expect a separate letter if somebody wants to voice significant critique

Does the article adequately reference differing views and opinions?

No

Are all factual statements correct, and are statements and arguments made adequately supported by citations?

Yes

Is the Open Letter written in accessible language?

Yes

Where applicable, are recommendations and next steps explained clearly for others to follow?

Yes

Is the rationale for the Open Letter provided in sufficient detail?

Yes

Reviewer Expertise:

climate change adaptation and health: global health

I confirm that I have read this submission and believe that I have an appropriate level of expertise to confirm that it is of an acceptable scientific standard.

# Reviewer report: Reviewer response for version 1

**DOI:** 10.21956/wellcomeopenres.25841.r117511

**Published:** 2025-02-11

**Authors:** Annabelle Workman, Arthur Wyns

**Affiliations:** 1 Melbourne Climate Futures, University of Melbourne, Melbourne, Australia; 2 University of Melbourne, Melbourne, Australia

This is a very timely, relevant and well-articulated project.

 -General comment: since none of the authors are affiliated with CCC or GLA, it would be good for the article to (i) clarify who in the CCC and GLA would be your main collaborators, (ii) confirm in writing that the CCC and GLA have agreed to collaborate on the project, and (iii) the role the CCC and GLA will play in each respective Work Package

 -General comment: the 5 research objectives could be more clearly linked to the 5 Work Packages. Currently the subchapters describing each work package describe the activities that will be undertaken, but do not clearly link back to the 5 main outputs/research objectives (at least not always or not in sufficient detail). Making these linkages more explicit in the subchapters on each work package will strengthen your argumentation.

 -General comment: Given the project’s focus on developing appropriate indicators with which to monitor the impact on health of climate mitigation and adaptation policies, it would be good to add a sub-chapter or a few paragraphs on the theoretical underpinnings of the health co-benefits of mitigation measures. It also seems to me to be a very significant step forward to go from - on the one hand - the published findings of the Pathfinder Initiative indicating a relative lack of indicators for health co-benefits in certain sectors, to - on the other hand - an extensive set of indicators across various sectors that can be incorporated into monitoring frameworks. It would be good to expand on how PAICE will manage to deliver this progression within the constraints of the project.

 -“In terms of climate targets, the UK has been a world leader”: I would suggest clarifying this sentence by adding a verb, i.e.  ‘In terms of setting climate targets’. As you rightfully point out further down, the UK should perhaps not be considered a champion when it comes to implementing climate targets.

 -“Acceleration of healthy, sustainable development”. Suggest adding ‘climate-resilient’ to this list, which is crucial especially in relation to climate and health.

 -“It requires a profound understanding of the system structure behind the lack of progress in implementation”: I would recommend leaving this sentence out, or as a minimum to provide further clarification. The current wording implies the lack of progress is caused solely by the inherent system in which society operates.

 -“ Evidence-based approaches to inform policy1 development and ultimately implementation”: Suggest adding ‘monitoring of progress’ as a third function here, especially given the objectives of the PAICE program.

 -“ anticipated impacts: Short-term”: given your objective to translate the research by working with the GLA (and the need for this research to take place first before it can be translated) would it be more appropriate to label the work with the GLA as medium-term or even long-term?

 -“ Underpinning principles: Academic-policy engagement”: this is a helpful theoretical overview. This paragraph would benefit from a few sentences on the way PAICE hopes to strengthen this academic-policy engagement (similarly to the first paragraph in your ‘Systems thinking’ subchapter). For example, how will you be working with the CCC? What will this engagement look like when you translate research with the GLA?

 -“ Underpinning principles: Transdisciplinary research”: When you describe ‘actors outside academia’, it might be worthwhile to point out that in the case of this project, that will include policymakers.

 - “WP2 will identify current and planned climate policy measures in the housing, energy, food and transport sectors, starting from the CCC monitoring maps”: could you please provide a reference or more information on what the CCC monitoring maps contain? As someone who is not very familiar with the CCC’s work, I currently struggle to fully understand the starting point of WP2 (ie what data is already available, versus what will have to be modelled).

 -"PAICE will also work with the GLA": this paragraph merits further detail as to how exactly the collaboration with the GLA will take place.

 -Background: Is there an opportunity to briefly expand on the challenges of evidence-based policy development and the impact on health (in)equity? For those not working in the climate and health space, it might not be clear that poorly designed policies can exacerbate and entrench existing health inequities, which is an important point as it underpins the rationale for using indicators and monitoring frameworks to evaluate and track progress.

 -Background: Is there an opportunity to briefly explain the siloed approach not only within adaptation and mitigation interventions (i.e. between sectors), but also between these two climate responses? This will help to substantiate the need for an integrate mitigation and adaptation monitoring framework.

 -Systems analysis (WP2): Who will the participatory workshops be held with to review cross-sector interactions and policy measures?

 -Evaluation (WP5): “Activities such as participatory workshops, interviews and observations will provide opportunities for reflection and collaborative learning…” Is there any mechanism through the GLA (or CCC) that involves representatives from marginalised or at-risk populations? I.e. is there an avenue for those who experience health inequities to input into the project in any way? Assuming community engagement is out of scope for the project, it might be worth flagging this – or clarifying if it is a distinct process undertaken by GLA/CCC via other means.

Does the article adequately reference differing views and opinions?

Yes

Are all factual statements correct, and are statements and arguments made adequately supported by citations?

Yes

Is the Open Letter written in accessible language?

Yes

Where applicable, are recommendations and next steps explained clearly for others to follow?

Partly

Is the rationale for the Open Letter provided in sufficient detail?

Yes

Reviewer Expertise:

climate change action; policy; health and health equity; transdisciplinary research

We confirm that we have read this submission and believe that we have an appropriate level of expertise to confirm that it is of an acceptable scientific standard, however we have significant reservations, as outlined above.
